# Healthcare Access and Experiences of Racial Discrimination as Predictors of General Vaccine Hesitancy

**DOI:** 10.3390/vaccines11020409

**Published:** 2023-02-10

**Authors:** Sheena CarlLee, Don E. Willis, Jennifer A. Andersen, Morgan Gurel-Headley, Shashank S. Kraleti, James P. Selig, Ramey Moore, Alexandra Diaz-Cruz, Michael D. Macechko, Pearl A. McElfish

**Affiliations:** 1 College of Medicine, University of Arkansas for Medical Sciences Northwest, Fayetteville, AR 72703, USA; swcarllee@uams.edu; 2 College of Medicine, University of Arkansas for Medical Sciences Northwest, Springdale, AR 72762, USA; dewillis@uams.edu (D.E.W.); jaandersen@uams.edu (J.A.A.); rameymoore@uams.edu (R.M.); mdmacechko@uams.edu (M.D.M.); 3 College of Medicine, University of Arkansas for Medical Sciences, Little Rock, AR 72205, USA; mpgurel@uams.edu (M.G.-H.); skraleti@uams.edu (S.S.K.); acruz@uams.edu (A.D.-C.); 4 Fay W. Boozman College of Public Health, University of Arkansas for Medical Sciences, Little Rock, AR 72205, USA; 5 Fay W. Boozman College of Public Health, University of Arkansas for Medical Sciences Northwest, Springdale, AR 72762, USA; jpselig@uams.edu

**Keywords:** vaccines, general vaccine hesitancy, healthcare access, racial discrimination

## Abstract

The literature regarding vaccine hesitancy is limited to specific vaccines rather than general vaccine hesitancy. No studies have examined the relationship of general vaccine hesitancy to healthcare access and experiences of racial discrimination. This study fills gaps by examining: (1) socio-demographic factors; (2) associations between healthcare access; and (3) experiences with racial discrimination and general vaccine hesitancy. Survey data were obtained from 2022 US adults from 7 September to 3 October 2021. Racial and ethnic minority populations were oversampled. Age, gender, race, and education were predictors of vaccine hesitancy. Asian respondents had less than two-thirds the odds of being vaccine hesitant. Healthcare access was associated with vaccine hesitancy. Not having health insurance coverage, not having a primary care provider, and not seeing a provider for a routine check-up in the past two years were associated with higher vaccine hesitancy. For every one-point increase in racial discrimination score (0–45), the odds of being more vaccine hesitant increased by a factor of 1.03. The findings demonstrate that policy, systems, and environmental factors are critical to addressing vaccine hesitancy. Given the associations between vaccine hesitancy and racial discrimination and healthcare access, more attention should be given to inequities in the healthcare systems in order to address vaccine hesitancy.

## 1. Introduction

The World Health Organization (WHO) defines vaccine hesitancy as a reluctance or refusal to vaccinate despite the availability of vaccines and declares it one of the top 10 threats to global health in 2019 [1]. Up to three million deaths are prevented each year because of vaccines, yet vaccine hesitancy has risen significantly, especially since the COVID-19 pandemic [1,2]. In the United States (US), pharmaceutical companies utilized unprecedented taxpayer investments to quickly develop an effective mRNA-based vaccine to combat the COVID-19 pandemic [3]. When the vaccine initially became available to the public and supplies were limited, the government took a stepwise approach to vaccine delivery, based on comorbid conditions and occupational exposure risk. Evidence suggests that many Americans agreed with this approach regarding the distribution of limited supply [4]. However, as the availability of vaccines has increased, the vaccination demand has decreased. COVID-19 vaccine hesitancy is a particularly divisive issue in the US, as studies have found that an individual’s choice to forgo vaccination is influenced by lack of trust in the Centers for Disease Control and Prevention, skepticism regarding the COVID-19 pandemic, and conservative political leaning. Americans raised questions about the safety of the vaccine, which was developed in record time, as well as the gravity of the COVID-19 infection [3]. In unprecedented numbers, Americans were turning to sources other than healthcare providers for sources of vaccine-related information [5]. This is in contrast to lower-income countries where there was significantly higher willingness for COVID-19 vaccination among the general population when compared to the US [6]. The challenge of vaccine hesitancy did not begin with the COVID-19 pandemic, yet the pandemic may have shifted or exacerbated the primary contributors to vaccine hesitancy. It is important to have a strong understanding of the contributors to general vaccine hesitancy which have arisen during this pandemic so that the medical community and policymakers can improve vaccine delivery in the future. As the prevalence of vaccine hesitancy increases, vaccine-preventable diseases become more likely to resurge, and their negative health outcomes increase.

Some groups, based on sociodemographic factors such as age, sex, race/ethnicity, or education, are more likely to report vaccine hesitancy. Prior research has shown participants who identify as women, are younger, and have lower educational attainment report greater vaccine hesitancy [2,7,8,9,10,11,12,13,14,15,16,17,18,19]. Research shows differences in hesitancy among some minority communities, with studies showing Black communities are more likely to report vaccine hesitancy [20,21,22], while Asian communities report less hesitancy; however, the results are sometimes mixed depending on how racial and ethnic groups are aggregated by researchers. Further, the body of research that looks beyond individual-level sociodemographic factors to examine policy, systems, and environmental factors (e.g., access to healthcare and experiences with racial discrimination) is limited [23,24,25,26,27,28]. Multiple studies have now documented positive associations between experiences of racial discrimination and vaccine hesitancy towards influenza and COVID-19 vaccines [23,24,27,28]. Given the evidence of historical and contemporary racism within both medical and non-medical institutions [29,30,31,32,33], it is critical that researchers open inquiries that can investigate the relationship between vaccine hesitancy and experiences of racial discrimination. 

The existing literature regarding policy, systems, and environmental factors and their effects on vaccine hesitancy is limited to specific vaccines such as COVID-19 or influenza, rather than general vaccine hesitancy [23,24,25,26,27]. This leaves a gap in knowledge about general vaccine hesitancy. No studies have been published examining the relationship of general vaccine hesitancy to healthcare access and experiences of racial discrimination. This study sought to fill these gaps in the literature with the following three aims. The first aim examined socio-demographic factors including sex, age, education, race/ethnicity, marital status, and general vaccine hesitancy. We hypothesized that, consistent with prior literature, those who identify as women, are younger, and have lower educational attainment would report greater general vaccine hesitancy. The second aim examined the associations between healthcare access and general vaccine hesitancy. We hypothesized that participants with health insurance coverage and a primary care provider would have lower general vaccine hesitancy and that those who have not seen a provider for a routine check-up in the past two years or have forgone medical care due to costs would have higher general vaccine hesitancy. The third aim examined lifetime experiences with racial discrimination and general vaccine hesitancy. We hypothesized that those who report more lifetime experiences of racial discrimination would have greater general vaccine hesitancy.

## 2. Methods

### 2.1. Procedures

Data were obtained through an online survey of 2022 US adults. Potential respondents were recruited by Atomik Research from an online opt-in panel of individuals from across the US between 7 September 2021 and 3 October 2021. Those who were eligible and completed the survey made up 25.1% of the total number of individuals who entered the online survey (8067). The overall conversion rate among eligible individuals was 62%. English and Spanish versions of the survey were available to respondents. Inclusion criteria included being aged 18 years or older and currently residing in the US. The following information about the study was provided to each potential respondent in recruitment emails: (1) estimated study duration (approximately 10 minutes); (2) potential risks and benefits of participation; (3) the voluntary nature of participation; and (4) confidentiality of participation and data. Respondents provided consent by agreeing to participate in the survey. Study procedures were reviewed and approved by the University of Arkansas for Medical Sciences Institutional Review Board (IRB #263020). 

Racial and ethnic minority populations, including Asian, Black, Hispanic/Latinx, American Indian or Alaska Native, and Native Hawaiian or Pacific Islander were intentionally oversampled. This was necessary to ensure a broad representation and to avoid the potential aggregation of data from racial and ethnic groups, which often conceals diverse attitudes and lived experiences [34,35]. The random iterative method [36] was used to weight data to be representative of the US population across demographic variables including gender (men, women, non-binary), race and ethnicity (Asian, Black, Hispanic/Latinx, American Indian or Alaska Native, and Native Hawaiian or Pacific, and White), and age (18–24, 25–34, 35–44, 45–54, 55–64, 65+). The 2019 US Census estimates were used to generate the weight [37].

### 2.2. Measures

#### 2.2.1. General Vaccine Hesitancy

The dependent variable was an ordinal measure of general vaccine hesitancy [38]. Respondents were asked: “Overall, how hesitant are you about getting vaccinations?” Response options included “not at all hesitant,” “a little hesitant,” “somewhat hesitant,” and “very hesitant”.

#### 2.2.2. Sociodemographic Characteristics

Age was estimated from respondents’ reported birth year and was categorized into four groups: 18–29, 30–44, 45–59, and 60+. Race/ethnicity was measured using standard race and ethnicity items from the Behavioral Risk Factor Surveillance System. Respondents were grouped into six categories: non-Hispanic Asian, non-Hispanic Black, Hispanic/Latinx, non-Hispanic American Indian or Alaska Native, non-Hispanic Native Hawaiian or Pacific Islander, and non-Hispanic White. Gender was reported as either man or woman. Although a third and fourth option of non-binary and self-defined were available, too few respondents selected these options (N = 3) to be included in the analysis. Respondents reported their highest level of school completed and were grouped into the following: high school degree or lower, some college/associate degree, and bachelor’s degree or higher. Marital status was collapsed into the following: married/coupled (married or member of an unmarried couple) and single/uncoupled (divorced, widowed, separated, or never married). 

#### 2.2.3. Experiences of Racial Discrimination

Lifetime experiences of racial discrimination were measured using an established 9-item measure [39]. Racial discrimination scores were calculated as the sum of the lifetime frequency of experiences of racial discrimination. Respondents were asked if they had “ever experienced discrimination, been prevented from doing something, or hassled or made to feel inferior in any of the following situations” due to their race, ethnicity, or color. Respondents provided the lifetime frequency of racial discrimination experienced across nine different social situations: (1) at school; (2) when being hired for a job; (3) at work; (4) in securing housing; (5) in receiving medical care; (6) at a store or restaurant; (7) when securing credit or applying for a mortgage; (8) on the street, in public; and (9) from police or by the courts. Response options included: (1) never; (2) once; (3) two or three times; and (4) four or more times. Responses were coded into three weighted categories: never = 0, two or three times = 2.5, and four or more times = 5. Racial discrimination scores were summed across the nine responses and could range from a minimum of 0 to a maximum of 45 [39]. The nine items had high internal consistency (α = 0.92).

#### 2.2.4. Healthcare Access

Healthcare access was measured using four survey items: (1) Do you have any kind of healthcare coverage, including health insurance, prepaid plans such as HMOs, government plans such as Medicare, or Indian Health Service? (Yes/No); (2) Do you have one person you think of as your personal doctor or healthcare provider? (Yes/No); (3) About how long has it been since you last visited a doctor for a routine check-up? Response options included: in the past year, in the past 2 years, in the past 5 years, 5 or more years ago, never. Due to changes in routine care-seeking behaviors due to the COVID-19 pandemic, responses were collapsed into two categories: within the past 2 years and more than 2 years ago; and (4) Was there a time in the past 12 months when you needed a doctor but could not see one because of the cost? (Yes/No).

### 2.3. Statistical Analyses

A total of 2022 respondents completed the survey. No duplicate records were identified. Respondents with incomplete responses (N = 75; 3.7%) were omitted from analyses, resulting in a final analytic sample of 1947 respondents. The most frequent missing item was healthcare coverage (N = 69; 3.4%). We present weighted and unweighted descriptive statistics of all available data and results of weighted ordinal logistic regression. The model predicts the odds of being more vaccine hesitant. We tested the proportional odds assumption using forward selection and setting the proportional odds criteria for each variable at >0.025 [40]. All variables except for healthcare coverage (*p* = 0.004) and routine doctor check-up (*p* = 0.004) met the equal slopes assumption. Thus, a partial proportional odds model was adopted, where the slopes of these two variables were allowed to differ across the levels of general vaccine hesitancy. Data were analyzed using SAS 9.4 (SAS Institute Inc., Cary, NC, USA).

## 3. Results

### 3.1. Descriptives

Table 1 provides the weighted percentages and unweighted frequencies among respondents with complete data (N = 1947). Some degree of hesitancy to vaccines was reported by the majority of respondents (51.9%), with 11.3% reporting they are “very hesitant” towards vaccines. Among healthcare access variables, the majority of respondents had healthcare coverage (87.0%), had a primary care provider (82.2%), and had a routine doctor check-up within the last two years (81.6%). Only 16.2% of respondents reported having to forgo needed healthcare due to cost in the past year. The mean racial discrimination score among respondents was 9.3 (±11.2).

### 3.2. Weighted Ordinal Logistic Regression

Table 2 provides the results of the partial proportional odds ordinal logistic regression based on the 1947 complete cases. The proportional odds assumption was relaxed for healthcare coverage and routine doctor check-up.

Age, gender, race, education, and racial discrimination were significant sociodemographic predictors of general vaccine hesitancy. Younger respondents (18–29 and 30–44 years) each had nearly twice the odds of the oldest respondents (60+ years) of being more vaccine hesitant [OR = 1.87(95%CI = 1.40, 2.50); OR = 1.80(95%CI = 1.41, 2.30), respectively]. Adults aged 45–59 years had 1.60 (95%CI = 1.25, 2.04) times greater odds of being more vaccine hesitant compared with the oldest respondents. Women had nearly twice the odds as men (OR = 1.79; 95%CI = 1.50, 2.14) of being more vaccine hesitant. Asian respondents had less than two-thirds (OR = 0.61; 95%CI = 0.44, 0.84) the odds of being more hesitant when compared to their White counterparts. No other significant differences were observed by race/ethnicity. Respondents with a high school diploma or less had 1.28 (95%CI = 1.02, 1.60) times greater odds of being more vaccine hesitant than respondents with a bachelor’s degree or higher. For every one-point increase in racial discrimination score, the odds of being more vaccine hesitant increased by a factor of 1.03 (95%CI = 1.02, 1.04).

Not having a primary care provider, forgoing healthcare due to cost, not having health insurance, and not having a routine check-up in the past two years were significant health access predictors of general vaccine hesitancy. Respondents without a primary care provider and those who had to forgo healthcare due to cost had approximately one and a third times greater odds [OR = 1.35(95%CI = 1.04, 1.75); OR = 1.29(95%CI = 1.01, 1.64), respectively] of being more vaccine hesitant compared with those who had a primary care provider and those who did not have to forgo healthcare due to cost.

Healthcare coverage status differed in effect across levels of general vaccine hesitancy. Insured and uninsured respondents had odds of being very hesitant compared with being not hesitant at all that were not significantly different (*p* = 0.577). Compared to uninsured respondents, insured respondents had approximately two-thirds the odds of being somewhat vaccine hesitant (OR = 0.68; 95%CI = 0.49, 0.93) and a little vaccine hesitant (OR = 0.65; 95%CI = 0.47, 0.90) compared with being not hesitant at all.

Routine doctor check-up frequency also differed in effect across levels of general vaccine hesitancy. Compared with respondents who had a routine check-up in the last two years, respondents whose most recent doctor check-up was more than two years ago consistently had greater odds of being very hesitant (OR = 2.90; 95%CI = 2.06, 4.09), somewhat hesitant (OR = 1.94; 95%CI = 1.46, 2.59), and a little hesitant (OR = 1.66; 95%CI = 1.25, 2.19) toward vaccines in general, compared with not being hesitant at all.

## 4. Discussion

This study examined general vaccine hesitancy by sociodemographic characteristics, healthcare access, and lifetime experiences of racial discrimination. The first hypothesis was partially supported. The analysis of sociodemographic factors revealed age, gender, race, and education were predictors of general vaccine hesitancy. Consistent with prior literature, younger respondents had nearly two times greater odds than the oldest respondents of being more vaccine hesitant [8,41]. Additionally, consistent with prior literature, women had greater odds of vaccine hesitancy than men [42,43,44], and those with a high school diploma or less had greater odds of vaccine hesitancy compared to those with higher educational attainment [2,7,16,45].

Results on race and ethnicity were mixed. Asian respondents had less than two-thirds the odds of being vaccine hesitant compared to their White respondents; no other significant differences were observed by race/ethnicity. The findings are consistent with prior studies, which have shown lower vaccine hesitancy among Asian individuals [22,46,47,48,49]. Although many studies have cited that Black respondents are more hesitant and less likely to be vaccinated, the research on vaccine hesitancy among Black and Hispanic populations has produced mixed results [21,22,26,38,45,49,50,51,52,53]. A possible explanation for this is that a high proportion of our study participants had a relationship with a primary care provider. Studies have shown that Black males who are in regular contact with a primary care provider or other healthcare clinic are less likely to be vaccine hesitant [54]. This study makes an essential contribution to the literature because it is one of the first national studies to have a large number of American Indian or Alaska Native participants. This study is also one of the first to have a large number of disaggregated Asian and Native Hawaiian or Pacific Islander participants. Given that differences were found between Asian and White respondents, but not between Native Hawaiian or Pacific Islander and White respondents, these results provide further evidence for the need to disaggregate data on Asian and Native Hawaiian or Pacific Islander individuals. The insufficient recruitment of individuals in these racial and ethnic groups leads, in turn, to the aggregation of groups and erasure of diversity of experiences and hesitancy towards vaccination.

The hypothesis that healthcare access would be associated with general vaccine hesitancy was supported. Having health insurance coverage, having a primary care provider, and having seen a provider for a routine check-up in the past two years were all associated with lower general vaccine hesitancy. Furthermore, there was an association between general vaccine hesitancy and having forgone medical care due to cost. These findings support the importance of improving access to primary care as a basic public health measure. Establishing an on-going relationship with a primary care provider allows for quick access to a trusted source of health-related information during times of uncertainty, such as the COVID-19 pandemic. These results add to the recent literature, suggesting the importance of healthcare provider relationships in COVID-19 and influenza vaccinations [45,53,55,56,57,58,59].

The hypothesis that experiences with racial discrimination would be associated with general vaccine hesitancy was supported. For every one-point increase in racial discrimination score (0–45), the odds of being more vaccine hesitant increased by a factor of 1.03. This is the first study to our knowledge that examines general vaccine hesitancy and experiences with racial discrimination. The findings are consistent with a small body of literature that has examined racial discrimination and COVID-19 vaccine hesitancy among Black adults [24], as well as other studies which have found an association between racial discrimination and hesitancy towards influenza [26,27] and COVID-19 vaccinations [28]. Importantly, this study is the only one of its kind to have a high proportion of American Indian or Alaska Native and Native Hawaiian or Pacific Islander respondents. These groups have suffered historical trauma [60,61,62,63,64,65,66], and the findings of this study are reflective of the impact of that trauma on vaccine hesitancy. The literature shows that outreach and connection in the community by healthcare providers correlates to vaccine uptake [67,68,69,70]. Understanding racial disparities in vaccine hesitancy within the broader context of racism (including but not limited to racial discrimination) is critical for work aiming to generate knowledge that may improve vaccination rates rather than victim-blame or perpetuate racial stereotypes. Reports of racial disparities in health or health behavior with absent considerations for the social context of racism may also reproduce myths of biological race [71].

### Strengths and Limitations

This study has several limitations. Cross-sectional data were used; therefore, we cannot make causal claims. Furthermore, we utilized an online survey, which limits accessibility for those without regular internet access, and the measures were self-reported. Self-reported measures provide many advantages but are also subject to social desirability bias. The study is strengthened by oversampling groups that are typically aggregated, allowing for the disaggregation of racial/ethnic groups such as Asian and Native Hawaiian or Pacific Islanders who are often combined. This is important in the literature on vaccine hesitancy because what is known about COVID-19 vaccine hesitancy among Asian and Native Hawaiian or Pacific Islander populations is often contradictory [72]. There is literature exploring the effects of religion and public health messaging on intentions to vaccinate [73,74]. While religion and public health messaging were not examined in this study, they may have influenced hesitancy. Although we have examined many important correlates of vaccine hesitancy, there are likely other unknown correlates which we have left unexamined. For example, while we have examined the relationship between general vaccine hesitancy and one measure of racism, we have not examined associations with measures of structural racism.

## 5. Conclusions

This is the first known study to examine experiences with racial discrimination and general vaccine hesitancy and one of the first studies to report the important association between healthcare access and general vaccine hesitancy. The findings demonstrate that policy, systems, and environmental factors such as racial discrimination, access to insurance, having a primary care provider, and routine primary care services are critical to addressing vaccine hesitancy. Based on the results from this study, it can be suggested that health policies which support improved access to primary care in communities with racial, ethnic, and socioeconomic diversity will have a positive impact on vaccination rates. Much of the current literature on vaccine hesitancy is focused on individuals’ attitudes and sociodemographic factors. Given the associations between vaccine hesitancy and racial discrimination and healthcare access, more attention needs to be given to addressing the inequity in US social and healthcare systems as a means of addressing vaccine hesitancy. Scholars and public health officials that want to address vaccine hesitancy must first acknowledge that vaccine hesitancy is often reasonable and justified among minoritized racial groups that experience historical and ongoing discrimination across society.

## Figures and Tables

**Table 1:** Weighted and unweighted descriptive statistics for all study variables (N = 1947).

Measures	Weighted % or Mean ± SD	Unweighted N
**Age**		
18–29	17.5	320
30–44	27.2	695
45–59	24.1	484
60+	31.2	448
**Gender**		
Men	50.0	921
Women	50.0	1026
**Race/Ethnicity**		
White	41.1	400
Black	19.8	389
Hispanic/Latinx	19.7	386
Asian	10.0	295
AIAN	4.7	241
NHPI	4.7	236
**Education**		
HS diploma/GED or lower	27.2	538
Some college/associate degree	34.4	675
Bachelor’s degree or higher	38.4	734
**Marital Status**		
Married/coupled	48.1	951
Unmarried/uncoupled	51.9	996
**Racial Discrimination Score**	9.3 ± 11.2	
**Healthcare Coverage**		
Yes	87.0	1671
No	13.1	276
**Primary Care Provider**		
Yes	82.2	1553
No	17.9	394
**No Medical Care Due to Cost**		
No	83.8	1580
Yes	16.2	367
**Routine Doctor Check-up**		
In the past 2 years or less	81.6	1573
More than 2 years ago	18.4	374
**General Vaccine Hesitancy**		
Not hesitant at all	48.1	880
A little hesitant	29.0	569
Somewhat hesitant	11.6	253
Very hesitant	11.3	245

Note: percentages may not total 100 due to rounding. SD = standard deviation; AIAN = American Indian or Alaska Native; NHPI = Native Hawaiian or Pacific Islander; HS = high school; GED = graduate equivalency degree.

**Table 2:** Weighted ordinal logistic regression—predictors of general vaccine hesitancy (N = 1947).

	Β	SE	*p*	OR (95% CI)
**Age**				
18–29	0.63	0.148	**<0.** **001**	1.87 (1.40, 2.50)
30–44	0.59	0.125	**<0.** **001**	1.80 (1.41, 2.30)
45–59	0.47	0.125	**<0.** **001**	1.60 (1.25, 2.04)
60+	—	—	—	—
**Gender**				
Women	0.58	0.091	**<0.** **001**	1.79 (1.50, 2.14)
Men	—	—	—	—
**Race/Ethnicity**				
Asian	–0.50	0.163	**0.002**	0.61 (0.44, 0.84)
Black	0.25	0.129	0.053	1.28 (0.997, 1.65)
Hispanic/Latinx	–0.20	0.129	0.128	0.82 (0.64, 1.06)
AIAN	0.31	0.213	0.146	1.36 (0.90, 2.07)
NHPI	–0.11	0.217	0.607	0.89 (0.58, 1.37)
White	—	—	—	—
**Education**				
HS diploma/GED or lower	0.25	0.115	**0.033**	1.28 (1.02, 1.60)
Some college/associate degree	0.20	0.106	0.054	1.23 (0.997, 1.51)
Bachelor’s degree or higher	—	—	—	—
**Marital Status**				
Married/coupled	0.10	0.091	0.261	1.11 (0.93, 1.32)
Unmarried/uncoupled	—	—	—	—
**Racial Discrimination Score**	0.031	0.005	**<** **0** **.** **001**	1.03 (1.02, 1.04)
**Healthcare Coverage ^a^**				
Insured, very hesitant	0.11	0.204	0.577	1.12 (0.75, 1.67)
Insured, somewhat hesitant	–0.39	0.162	**0.015**	0.68 (0.49, 0.93)
Insured, a little hesitant	–0.43	0.164	**0.009**	0.65 (0.47, 0.90)
Uninsured, not hesitant at all	—	—	—	—
**Primary Care Provider**				
No	0.30	0.133	**0.025**	1.35 (1.04, 1.75)
Yes	—	—	—	—
**No Medical Care Due to Cost**				
Yes	0.25	0.122	**0.038**	1.29 (1.01, 1.64)
No	—	—	—	—
**Routine Doctor** **Check-up ^a^**				
>2 years ago, very hesitant	1.06	0.175	**<** **0** **.** **001**	2.90 (2.06, 4.09)
>2 years ago, somewhat hesitant	0.66	0.146	**<** **0** **.** **001**	1.94 (1.46, 2.59)
>2 years ago, a little hesitant	0.50	0.142	**<** **0** **.** **001**	1.66 (1.25, 2.19)
<2 years ago, not hesitant at all	—	—	—	—

Note: significant *p*-values are in **bold**. *Β* = beta; SE = standard error; OR = odds ratio; CI = confidence interval; AIAN = American Indian or Alaska Native; NHPI = Native Hawaiian or Pacific Islander; HS = high school, GED = graduate equivalency degree. ^a^ Healthcare coverage and routine doctor check-up failed to meet the equal slopes assumption. Thus, a partial proportional odds model was used, where the slopes of these two variables were allowed to differ across the levels of general vaccine hesitancy.

## Data Availability

The deidentified data underlying the results presented in this study may be made available upon request from the corresponding author, Pearl A. McElfish, at pamcelfish@uams.edu. The data are not publicly available in accordance with funding requirements and participant privacy.
